# Probing lysosomal activity *in vivo*

**DOI:** 10.52601/bpr.2021.200047

**Published:** 2021-02-28

**Authors:** Xin Li, Yanan Sun, Xiaochen Wang

**Affiliations:** 1 National Laboratory of Biomacromolecules, CAS Center for Excellence in Biomacromolecules, Institute of Biophysics, Chinese Academy of Science, Beijing 100101, China; 2 College of Life Sciences, University of Chinese Academy of Sciences, Beijing 100049, China

**Keywords:** Lysosomes, *C. elegans*, Maturation, Acidification, Cleavage activity

## Abstract

Lysosomes are membrane-bound organelles for biomolecule degradation and recycling. They also serve as a nutrient sensing and signaling center to maintain cell and tissue homeostasis. Lysosomal properties alter in response to developmental or environmental cues, but these changes are hard to track *in vivo*. Employing *C. elegans* as a model system, we have developed assays to examine and quantify lysosome properties *in vivo*, including lysosome maturation, acidification and cleavage activity. These assays can be used to reveal alterations of lysosomal activity during *C. elegans* development and in stress conditions.

## INTRODUCTION

Lysosomes are membrane-bound organelles for degradation and recycling of biomolecules. Cargos derived from multiple trafficking routes are sent to lysosomes for digestion (Cullen and Steinberg 2018). The resulting catabolites are reused for cell growth (Gonzalez *et al*. 2020). Lysosomes also serve as a signaling center for nutrient sensing and thus the maintenance of cell homeostasis (Lawrence and Zoncu 2019). mTOR and AMPK are activated on the lysosome surface under different conditions to regulate downstream signaling pathways (Gonzalez *et al*. 2020).

Lysosome was discovered by the biochemist Dr. de Duve in the 1950s, starting from his group’s study of a hexose phosphatase. They noted that an acid phosphotase, mainly as a control for enzyme activity measurement, was less active when using a mild homogenization process compared to a drastic homogenization. Then, they found out that it worked under pH 5 and its enzymatic activities were associated with a specific fraction of membrane surrounded particles. Later, four other acidic hydrolases were found to be enclosed within the same subcellular compartment, *i.e*. lysosome (de Duve 2005; Sabatini and Adesnik 2013). As the degradative organelle in cells, lysosomes contain over 60 hydrolases, which work in an acidic environment (pH 4.5–5.5). Lysosomes integrate and receive substrates from autophagic, endocytic and phagocytic pathways. The substrates of lysosomal hydrolases consist of various biomolecules including proteins, lipids, glycogen, and nucleic acids, which are degraded in the lysosomal lumen.

After degradation, the resulting catabolites are transported out of lysosomes by various membrane transporters and reused as building blocks for cell growth. Some of the catabolites can function as signaling molecules to regulate activity of the mTORC complex (Lawrence and Zoncu 2019). Lysosomal transporters include solute transporters that mediate ions flux across lysosome membranes, and transporters specific for amino acids, lipids and nucleotides (Huizing and Gahl 2020; Xiong and Zhu 2016). For example, V-ATPase is a proton pump responsible for maintaining the acidic lumen of lysosomes. We found that lysosomal activity is greatly promoted at the *C. elegans* molting stage by upregulation of the V-ATPase gene expression (Miao *et al*. 2020). Moreover, amino acid transporters are important for maintaining lysosome function and homeostasis (Gan *et al*. 2019; Liu *et al*. 2012). Defects in lysosomal acidity, hydrolases or membrane transporters cause substrate accumulation in the lysosomal lumen, which may lead to lysosome storage diseases (Liu *et al*. 2012, 2018; Nixon 2016; Parkinson-Lawrence *et al*. 2010).

Abnormal lysosome activity can be detected by biochemical approaches such as measurement of substrate amounts and/or enzyme digestion activity in purified lysosome fraction. To better appreciate the aberrations of lysosomal function *in vivo*, instead, we sought to investigate lysosome function and regulation in the model organism *C. elegans* (Li *et al*. 2016; Liu *et al*. 2012, 2018; Miao *et al*. 2020; Sun *et al*. 2020). Importantly, lysosomes exhibit variable morphological patterns in different tissues, at different developmental stages and during aging process in *C. elegans* (Li *et al*. 2016; Miao *et al*. 2020; Sun *et al*. 2020). Here, we present protocols for assaying lysosome activity in *C. elegans*. Particularly, we develop fluorescent reporters to trace lysosome maturation and acidification and we utilize western blot analysis to determine substrate cleavage, which indicates lysosomal degradation activity.

## MATERIALS AND EQUIPMENT

### Strains

Strains of *C. elegans* are cultured and maintained using standard protocols (Brenner 1974). The N2 Bristol strain is used as the wild-type strain. Standard microinjection methods were used to generate transgenic animals carrying extrachromosomal arrays (qxEx). Genome-integrated arrays (qxIs) were acquired by γ-irradiation to achieve stable expression from arrays with low copy numbers. The following strains were used: N2, *daf-2(e1370)*, *cup-5*(*bp510*), *qxIs257* (P*_ced-1_*NUC-1::CHERRY), *qxIs612* (P*_hs_*NUC-1::sfGFP::CHERRY), and *qxIs750* (P*_hs_*NUC-1::pHTomato).

### Buffers

• M9 (1 L): KH_2_PO_4_ 3 g, Na_2_HPO_4_ 6 g, NaCl 5 g, add ddH_2_O to 1 L, then add 1 mL 1 mol/L MgSO_4_ after autoclave;

• SDS-PAGE sample buffer (Sangon).

### Equipment

• Confocal microscope: LSM 880 Meta plus Zeiss Axiovert zoom (Carl Zeiss) with 488 (emission filter BP 503–530) and 543 (emission filter BP 560–615) lasers;

• Chemi-luminescence detection system (CLiNX Science instruments).

### Software

• LSM Image Browser and ZEN software (Carl Zeiss Inc.);

• Volocity (PerkinElmer);

• Image J;

• GraphPad Prism (GraphPad Software).

## OVERVIEW OF THE EXPERIMENTAL DESIGN

This protocol includes three sections. The first section provides a detailed procedure to track lysosomal maturation and acidification. The second section presents the steps for detecting lysosomal acidity, and the last section describes the method to measure lysosomal degradation activity.

**Figure 1:**
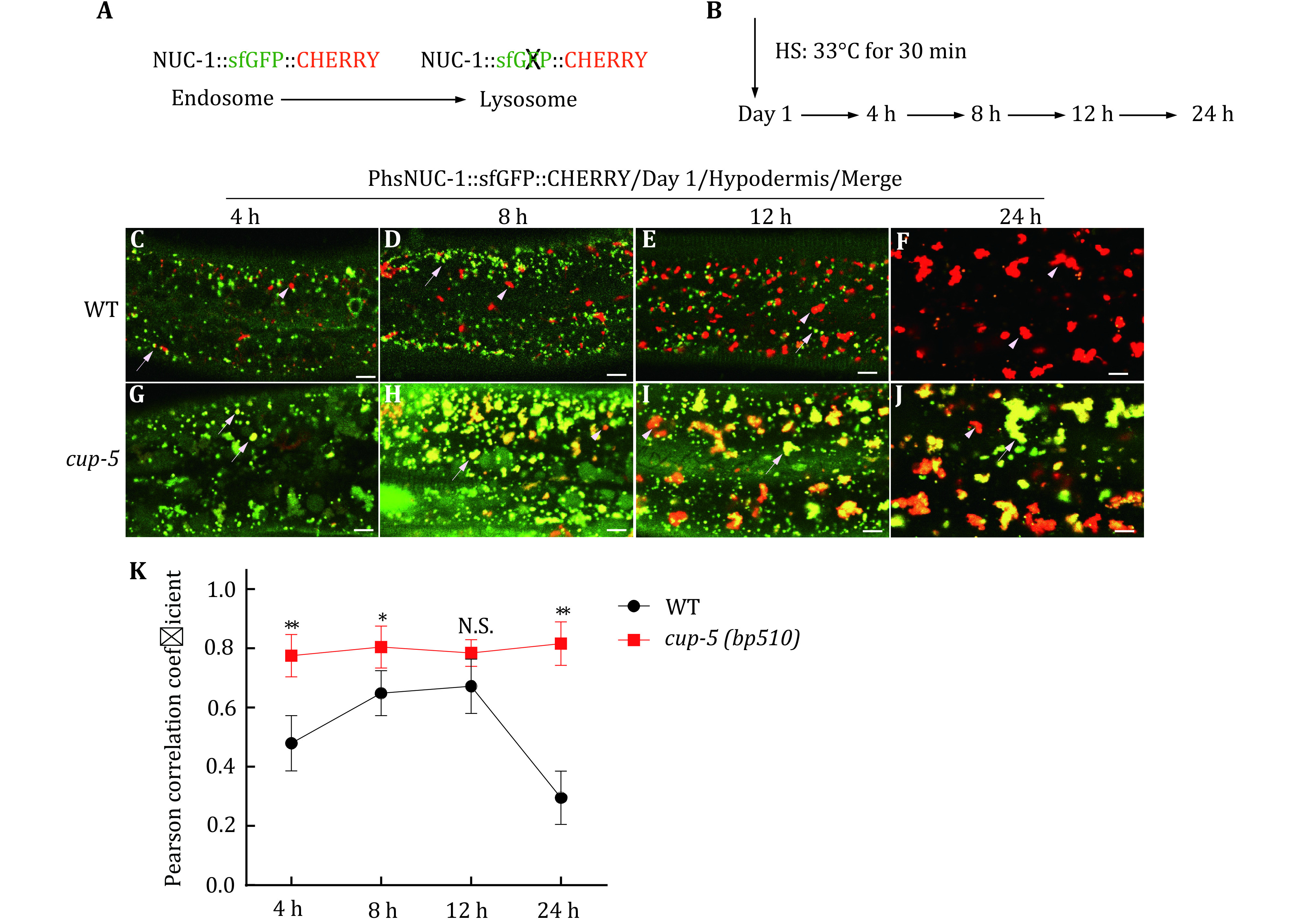
Lysosome maturation assay. **A**, **B** Diagram of the assay to examine lysosome maturation in the hypodermis using heat-shock (HS) induction of NUC-1::sfGFP::CHERRY. **C**–**J** Merged confocal fluorescence images of the hypodermis in wild type (WT) and *cup-5(bp510)* expressing NUC-1::sfGFP::CHERRY at 4, 8, 12 and 24 h post HS. White arrowheads indicate structures labeled by CHERRY only and white arrows indicate overlap of GFP and CHERRY. **K** Overlap of GFP and CHERRY is quantified by Pearson’s correlation coefficient. At least five animals were scored at each time point in each strain. Data are shown as mean ± SD. Two-way ANOVA with Tukey’s multiple comparison test was used to compare mutant datasets with the wild type. *p < 0.05, **p < 0.01; N.S.: no significance. Scale bars: 5 μm

In the first part of the protocol, we express NUC-1::sfGFP::CHERRY transiently using the heat-shock (hs) promoter in worms and we follow the delivery of the tandem fusion protein to lysosomes in the hypodermis at different time points (Fig. 1A, B). The GFP fluorescence is visible in endosomes but is quenched in lysosomes due to the acidic environment, whereas CHERRY fluorescence is visible in both compartments (Fig. 1A). Lysosomal maturation is indicated by gradual disappearance of GFP in the CHERRY-positive structures. We quantify colocalization of GFP and CHERRY using Volocity software in wild type and lysosome-defective *cup-5* mutants.

In the second part, we analyze lysosomal acidity. We fused the pH-sensitive fluorescent protein pHTomato with NUC-1, and we transiently express NUC-1::pHTomato using the heat-shock (hs) promoter. pHTomato has a p*K*a close to 7.8 and thus exhibits increased fluorescence when the pH is increased (Li and Tsien 2012). We measure pHTomato fluorescence intensity in each lysosome using Volocity software. Loss of the lysosomal Ca^2+^ channel CUP-5 impairs lysosome activity and acidity (Hersh *et al*. 2002; Miao *et al*. 2020; Sun *et al*. 2011; Treusch *et al*. 2004), whereas lysosomal acidity increases in the long-lived *daf-2* mutant worms (Sun *et al*. 2020). We assay the lysosomal acidity in wild-type, *cup-5* and *daf-2* worms.

In the third part, the lysosomal degradation activity is analyzed by examining cleavage of the NUC-1::CHERRY fusion protein and CPL-1 processing. CHERRY is cleaved from the NUC-1::CHERRY fusion protein by proteases in lysosomes, and the amount of CHERRY protein is visualized by western blot and quantified to indicate the degradation activity of lysosomes (Miao *et al*. 2020; Sun *et al*. 2020). Cathepsin L (CPL-1) is synthesized as an inactive pro-enzyme, which is converted to the active mature form in lysosomes through proteolytic removal of the pro-domain (Stoka *et al*. 2016). The processing of endogenous CPL-1 can be examined by western blot and quantified to indicate the degradation activity of lysosomes. These assays are performed in wild-type, *cup-5* and *daf-2* worms.

## STEP-BY-STEP PROCEDURE

### Lysosome acidification and maturation assay

(1) Worms are synchronized to the L4 stage and transferred to fresh NGM plates containing OP50. Worms are further cultured for 24 h to reach day 1 of adulthood.

(2) Worms at day 1 of adulthood are heat-shocked at 33 °C for 30 min to induce transient expression of NUC-1::sfGFP::CHERRY.

(3) The worms are then incubated at 20 °C for 4, 8, 12 and 24 h (Fig. 1B). At each time-point, worms are picked, and the hypodermis is imaged by confocal fluorescence microscopy (Fig. 1C–J).

(4) The colocalization of CHERRY and GFP is presented as the Pearson correlation coefficient score, which is calculated from the images by Volocity software (PerkinElmer). At least five worms are analyzed in each strain at each time point.

We found that the fluorescence of GFP, but not CHERRY, reduced gradually and disappeared completely at 24-h post heat-shock (HS) treatment. In wild type, the Pearson correlation coefficient increased at 8-h post HS and reduced after 12-h post HS (Fig. 1K). This indicates a gradual maturation of lysosomes in the *C. elegans* hypodermis. In *cup-5*, lysosomal acidification was impaired and the GFP signal was visible even at 24-h post HS (Fig. 1G–K). In our previous study, we used this assay to examine lysosome maturation during *C. elegan*s larval development (Miao *et al*. 2020). We found that GFP disappeared faster at molt compared with the intermolt stage, which suggests that the speed of lysosome maturation increases during molting (Miao *et al*. 2020).

### Lysosome acidity assay

(1) Gravid adult worms are bleached, and the released embryos are placed in M9 buffer for 24 h to reach the larvae 1 (L1) stage. The synchronized L1 worms are transferred to NGM plates containing OP50 and grown to adults.

(2) *C. elegans* adults (1-day post L4) expressing P*_hs_*NUC-1::pHTomato are collected and heat shocked at 33 °C for 30 min.

(3) Worms are incubated at 20 °C for 24 h before examination in order to let the fusion protein enter into lysosomes.

(4) Worms are examined by fluorescence microscopy. The average intensity of pHTomato per lysosome in the hypodermis is measured by Volocity software. At least five worms and over 200 lysosomes in each worm are analyzed in each strain.

(5) Raw data is loaded and analyzed in Graphpad. The paired *t*-test is performed to compare different datasets.

**Figure 2:**
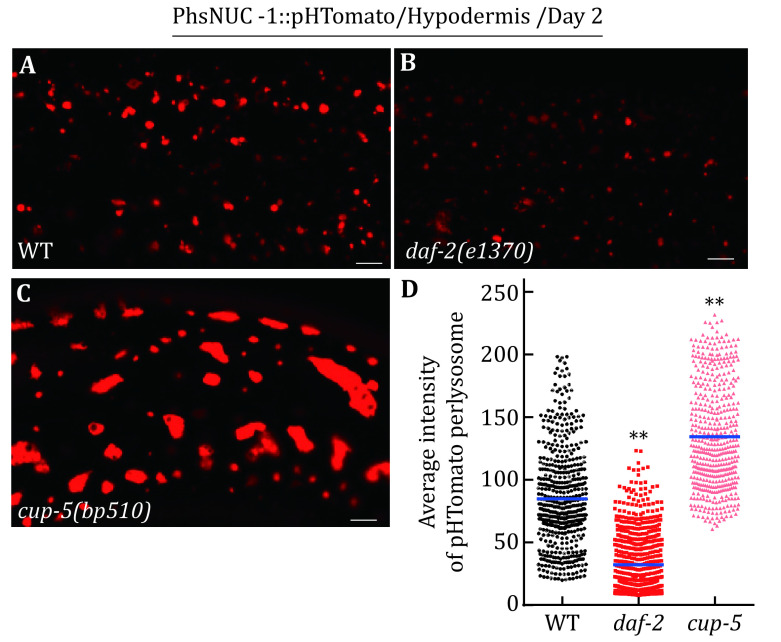
Lysosome acidity assay. **A**–**C** Confocal fluorescence images of the hypodermis at adult day 2 in wild type (WT), *daf-2(e1370)* and *cup-5(bp510)* expressing NUC-1::pHTomato controlled by the heat-shock (hs) promoter. Scale bars: 5 μm. **D** The average intensity of pHTomato per lysosome is quantified. At least 20 animals were scored in each strain. Data are shown as mean ± SD. The paired *t*-test was performed to compare the mutant datasets with the wild type. **p < 0.001

We performed this assay in wild-type, *cup-5* and *daf-2* worms (Fig. 2). We found that pHTomato intensity increased significantly in *cup-5* lysosomes but decreased in *daf-2* lysosomes (Fig. 2D). This indicates that lysosomal acidity is increased in *daf-2* worms but reduced in *cup-5* mutants.

**[CRITICAL STEP]** It is important that same laser power and exposure time are used to capture images of lysosomes in different strains. It is recommended to image different groups of samples at the same time. The average fluorescence intensity in multiple individual lysosomes (>200) is quantified to compare the different strains.

### Lysosome degradation activity assay

#### Quantification of NUC-1::CHERRY cleavage

(1) Worms are synchronized and cultured at 20 °C as described in the section of Lysosome acidity assay.

(2) Over 50 adults (1-day post L4) are collected and washed three times with M9 buffer to remove OP50 as much as possible.

(3) The worms are lysed in sample buffer by 2–3 rounds of freezing and boiling treatment. The resulting worm lysate is centrifuged at 14,000 r/min for 10 min. The supernatant is resolved by SDS-PAGE. NUC-1::CHERRY and CHERRY are detected by anti-CHERRY antibodies (SUNGENE BIOTECH, China, 1:1000). α-tubulin antibody (Sigma) is used at 1:5000 as an internal control.

(4) The amount of NUC-1::CHERRY and CHERRY is quantified by determining the total intensity of NUC-1::CHERRY and CHERRY bands using Image J software. CHERRY cleavage is calculated by dividing the amount of CHERRY by the total amount of NUC-1::CHERRY and CHERRY. At least three independent experiments are performed and analyzed.

#### Examination and quantification of CPL-1 processing

(1) Worm synchronization and sample preparation are done as described in the above section. The resulting worm lysate is analyzed by Western blot and CPL-1 is detected by anti-CPL antibodies (Antibody core, NIBS, 1:1000). α-tubulin antibody (Sigma) is used at 1:5000 as an internal control.

(2) The band intensity of the mature (lower band) and pro- (higher band) forms of CPL-1 is quantified by Image J software. CPL-1 processing is calculated by dividing the amount of mature CPL-1 by the amount of total CPL-1 (both pro- and mature forms). At least three independent experiments are performed and analyzed.

**Figure 3:**
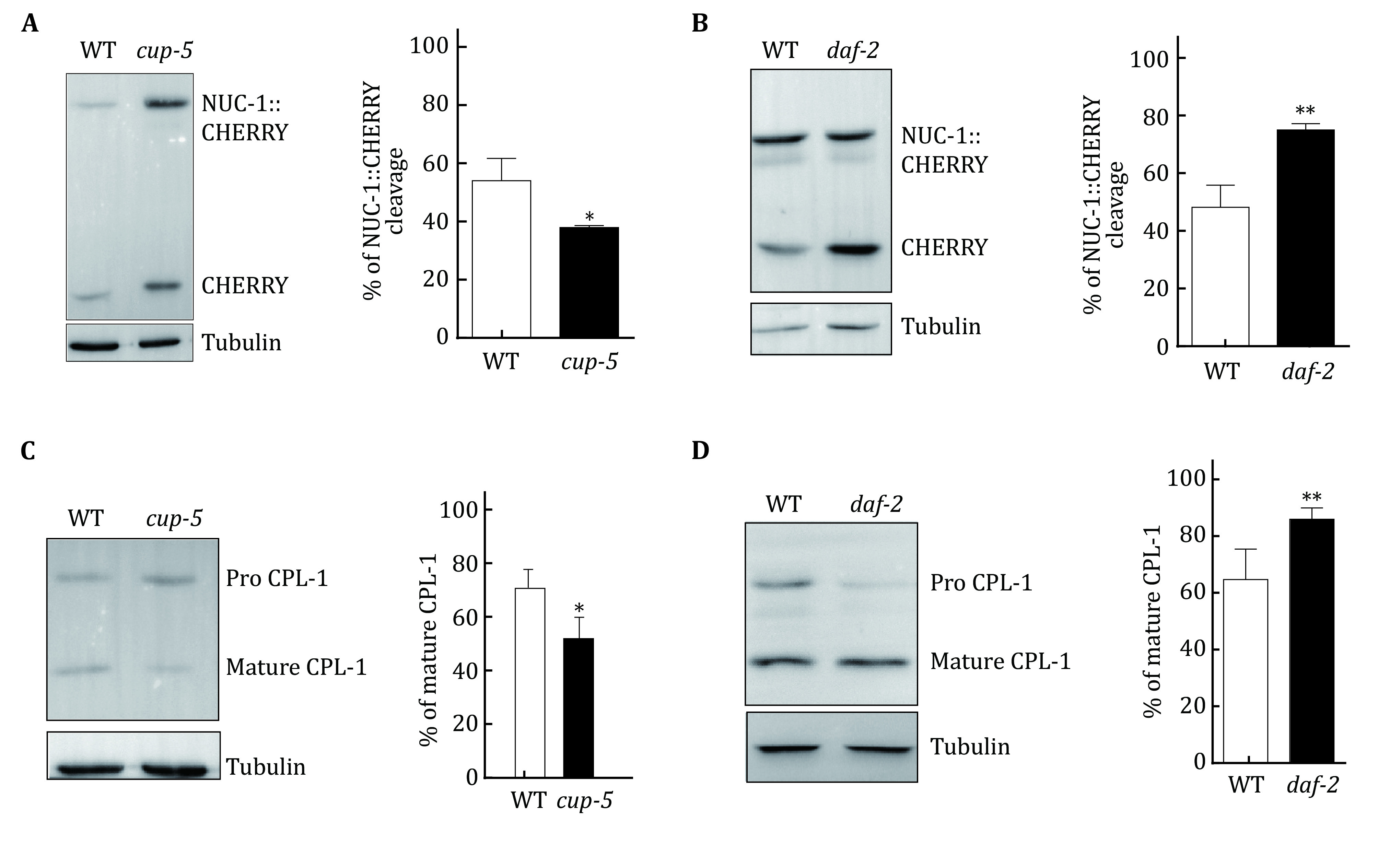
Lysosome degradation activity assay. **A**, **B** Western blot analysis of NUC-1::CHERRY cleavage in wild type (WT), *cup-5*(*bp510*) and *daf-2*(*e1370*) at day 1 of adulthood. The percentage of cleaved CHERRY was quantified and is shown in the right panels. **C**, **D** Western blot analysis of CPL-1 processing in wild type (WT), *cup-5*(*bp510*) and *daf-2*(*e1370*) at day 1 of adulthood. The percentage of mature CPL-1 was quantified and is shown in the right panels. Three independent experiments were performed. Data are shown as mean ± SD. The paired *t*-test was performed to compare the mutant datasets with the wild type. *p < 0.05, **p < 0.001

We used the assays detailed above to examine lysosome activity in wild-type, *cup-5(bp510)* and *daf-2(e1370)* worms. Notably, CHERRY cleavage reduced significantly in *cup-5* mutants but increased in *daf-2* worms compared with wild-type animals (Fig 3A, B). Similarly, CPL-1 processing was reduced significantly in *cup-5* mutants but increased in *daf-2* worms (Fig. 3C, D). These results suggest that lysosomal degradation activity is increased in *daf-2* but impaired in *cup-5* mutants.

**[CRITICAL STEP]** The bands resolved by Western blot should not be overexposed. For different antibodies or different protein abundance, the exposure time should be tested and optimized. In each experiment, equal amounts of total proteins are loaded, which are normalized by the levels of tubulin.

## FUTURE PERSPECTIVES

Lysosome maturation, acidity and degradation activity are important properties that can be monitored in combination to indicate the overall functionality of lysosomes in various biological processes. In this study, we provide detailed methods to assay lysosomal maturation, acidity and degradation activity in *C. elegans*. It is worth noting that in our study, lysosomal maturation and acidity assays are mainly performed in the hypodermis, a multi-nuclear syncytium that extends throughout most of the animal. As heat-shock treatment induces transient expression of reporters in multiple *C. elegans* tissues, these assays can be performed in other tissues if they are accessible to microscopy imaging. Moreover, lysosomal maturation and acidity assays can be performed in both larval and adult stages. However, we have not examined lysosomal acidity and maturation in small-sized cells such as neurons or embryonic cells. More sophisticated methods may be developed to examine lysosome property in small cells.

## Conflict of interest

Xin Li, Yanan Sun and Xiaochen Wang declare that they have no conflict of interest.
